# A Randomized Double-Blind Study Evaluating Intraperitoneal Ropivacaine Nebulization With and Without Nalbuphine for Post-operative Analgesia in Laparoscopic Cholecystectomy

**DOI:** 10.5152/TJAR.2022.21108

**Published:** 2022-06-01

**Authors:** Abullais R. Gowda, Nishith Govil, Ajit Kumar, Deepak Singla, Mridul Dhar, Farhanul Huda

**Affiliations:** 1 Department of Anaesthesiology, All India Institute of Medical Sciences, Rishikesh, India; 2 Department of General Surgery, All India Institute of Medical Sciences, Rishikesh, India

**Keywords:** Intraperitoneal nebulization, laparoscopic cholecystectomy, nalbuphine, numerical rating pain scale, postoperative pain, ropivacaine

## Abstract

**Objective:**

Local anaesthetics administered into the peritoneal cavity have been successfully used for post-operative pain relief in minimally invasive laparoscopic procedures. We intended to study and compare nebulized intraperitoneal ropivacaine with and without nalbuphine, with a placebo for post-operative pain relief in these surgeries.

**Methods:**

A prospective, randomized double-blinded study was conducted over a period of 1 year after institutional ethical clearance, in patients undergoing elective laparoscopic cholecystectomy. Subjects were randomized into 3 groups (S: saline, R: ropivacaine, RN: ropivacaine plus nalbuphine). The pain was assessed in the post-operative period using NRS scores (up to 24 hours). Kruskal-Wallis test was used for comparison, *P* < .05 was considered significant. Time to first rescue analgesia, total opioid requirement, and side effects were also recorded.

**Results:**

Groups were similar in terms of demographic data. Patients in the placebo group reported higher NRS scores than the other 2 study groups till 4 hours post-operative (earlier rescue analgesia). The addition of nalbuphine did not cause any statistically significant improvement in post-operative pain relief (NRS) as compared to ropivacaine administered alone. Intraperitoneal ropivacaine nebulization had no significant adverse effect as compared to placebo.

**Conclusions:**

Ropivacaine nebulization with or without nalbuphine is more effective than placebo for post-operative pain relief after laparoscopic cholecystectomy without significant side effects. Addition of nalbuphine to ropivacaine nebulization does not significantly improve pain relief after laparoscopic cholecystectomy.

**Main Points**

- The aim was to compare nebulized intraperitoneal ropivacaine with and without nalbuphine, with placebo for post-operative pain relief in laparoscopic cholecystectomies.
- Patients in the placebo group reported higher pain scores than in nebulized ropivacaine groups.
- Addition of nalbuphine did not cause any statistically significant improvement in post-operative pain relief as compared to ropivacaine alone.
- No significant side effects were observed with nebulization with ropivacaine.

## Introduction

Acute pain management after surgery is extremely essential. If not controlled adequately, it can worsen overall post-operative (PO) outcomes. The main aim of PO pain management is effective pain relief at appropriate doses of drugs, with minimal adverse effects. In laparoscopic cholecystectomy (LC), adequate control of PO pain helps in early mobility and patient discharge from the hospital, thus better patient satisfaction.^1,2^ Pain in laparoscopic abdominal surgeries is due to 3 components: somatic (port site incision), visceral (peritoneal), and phrenic nerve irritation (shoulder tip pain).^3,4^

Different modes of PO pain management options include opioids delivered intravenously (iv) or along with local anaesthetics in the form of Transversus Abdominis Plane block, epidural space blocks, peripheral nerve blocks, and nonsteroidal anti-inflammatory drugs given through various routes. Opioids may cause nausea, vomiting, respiratory depression, sedation, delayed return of gastrointestinal motility, and so on given iv.^5^

Nebulized drug delivery has been shown to have a better and uniform spread of local anaesthetic drugs compared to conventional drug instillation into the peritoneal cavity.^6^ Different types of nebulization devices have been used for pain relief after laparoscopic surgeries. Earlier studies have used cumbersome custom-made devices and devices that worked on hot evaporation-based nebulization principle.^7^ The objective of the present study is to assess the efficacy of intraperitoneal ropivacaine nebulization with and without nalbuphine on PO pain relief in LC compared to placebo, using a high-frequency ultrasonic nebulizer at the end of the procedure.

### Methods

A prospective, randomized double-blinded study was conducted after institutional ethical clearance and registered under clinical registry CTRI/2018/09/015764. Informed written consent was obtained prior to enrolment of patients as study participants. Patients were enrolled for 12 months, and the study was concluded in 1.5 years. The manuscript was prepared in accordance with CONSORT guidelines for randomized studies. Inclusion criteria were all patients aged 18-70 years of either sex, belonging to ASA physical status I and II being planned for LC and free from any pain in PO period (apart from biliary colic). Exclusion criteria were refusal by patient, conversion of laparoscopic approach to open surgery, duration more than 1 hour, the requirement of extra opioid above initial bolus intraoperatively, alcohol use or history of drug addiction, hepatic or renal impairment, allergy to the drugs being studied, any pre-existing source of abdominal pain apart from biliary colic, patients with communication problems, cognitive impairment, pregnancy or lactation, chronic pain treatment involving opioids, anti-epileptic therapy (to avoid confusion with seizures of local anaesthetic systemic toxicity [LAST]).

**Figure 1.**
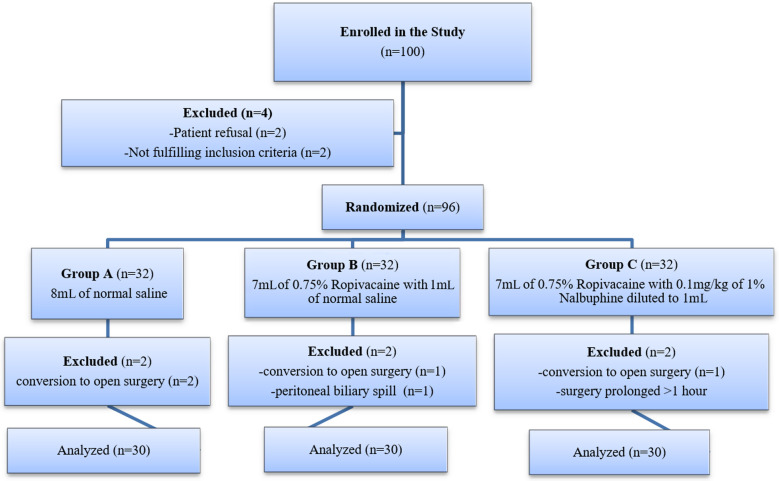
CONSORT algorithm showing study groups of patients with number of enrolments.

Randomization of patients into 3 groups was done by generating a table of random numbers and asking the patient to choose a number. Allocation concealment was done by the sealed envelope technique using opaque envelopes. This was done by the principal investigator who was not blinded. Group S: 8 mL of normal saline (placebo), Group R: 7 mL of 0.75% ropivacaine with 1 mL of normal saline, Group RN: 7 mL of 0.75% ropivacaine with 0.1 mg kg-1 of 1% nalbuphine diluted to 1 mL. All 3 groups had a total injection volume of 8 mL to maintain blinding at the level of intervention provider. The final effective concentration of ropivacaine in group R and RN was 0.65%. Reference studies have used drug volumes varying from 4 to 10 mL (0.75%) of ropivacaine.^2,8^ The dose and volume of the drug used in the current study were well below the toxic limit. Surgery was performed by surgeons with at least 5 years of experience, in all 3 groups. After measuring baseline hemodynamic parameters, anaesthesia induction was done using the following drugs: propofol 2 mg kg-1 iv, fentanyl 2 μg kg-1, and vecuronium 0.1 mg kg-1 iv. Maintenance was with sevoflurane 1.5-2% (in 50% O_2_ + 50% N_2_O). Cases in which surgery extended more than 1 hour or required additional fentanyl intraoperatively were excluded from analysis (Figure 1). Pneumoperitoneum was achieved with non-humidified CO_2_ and an intraabdominal pressure of 10-12 mm Hg was maintained intraoperatively in all subjects.

The patients were taught the use of a 0-10 numerical rating scale (NRS) on which 0 represents “no pain” and 10 represents the “worst possible pain.” The research assistant was not allowed inside the operation theater until the study drug solution was ready, in order to maintain blinding. Toward the end of surgery, the drug selected (based on group allocation) was nebulized with the help of Aerogen-Pro^®^ ultrasonic aerosol delivery device before the removal of trocar. The nebulization unit was in series with the insufflator device (between the device and insufflator tubing) and the nebulized drug was delivered through the tubing and trocar into the peritoneal cavity. Nebulization was done till the completion of 8 mL of drug solution (about 10 minutes). The CO_2_ gas was carefully evacuated from the peritoneal cavity at the end of surgery with open trocars. In all groups, port insertion sites were infiltrated with local anaesthetic solution (0.25% bupivacaine, 2 mL around each port site). Neuro-muscular blockade was reversed with neostigmine and glycopyrrolate using objective neuro-muscular monitoring (a train of 4 ratio greater than 0.9) uniformly in all groups. Paracetamol 1 g iv QID was given to all patients for first the 24 hours post-operatively.

The degree of PO pain was assessed using the NRS at 0, 1, 4, 8, 12, and 24 hours post-operatively along with hemodynamic parameters. This assessment was performed by a resident doctor who was blinded to the group allocation and was familiar with the use and interpretation of the NRS. Those patients with NRS > 4 at any of these recordings were administered a bolus of inj. fentanyl 1 μg kg-1 slow iv as rescue analgesia. Subsequent dose of fentanyl if required was not repeated before 4 hours of the previous dose.^9^ Second level of rescue analgesia was in the form of slow iv diclofenac 75 mg which was given slow iv over 10 minutes if persistent NRS >4 even after 15 minutes of fentanyl injection.^10^ Incidence of PO nausea and vomiting (PONV) was recorded; such a complaint was treated by ondansetron (4 mg iv). Time to first opioid requirement, total opioid consumption in the first 24 hours post-operatively, and occurrence of adverse events were recorded.

Primary objective of this study was to evaluate the efficacy of ropivacaine nebulization (with and without nalbuphine) compared to no drug or placebo in the reduction of PO pain in patients after LC. The primary outcome was NRS score at 1-hour PO. Sample size was calculated to be 87 patients (29 in each group), on the basis of 2 previous studies to be able to detect a difference in Pain Score by a scale of 2/10 at 1-hour PO between the study and placebo group.^6,11^ Assuming an alpha error of 5% and power of study being 80%. A final number of 100 was selected to compensate for exclusions. Secondary objectives and outcomes were to assess the incidence of side effects, total opioid consumption, and time to first rescue analgesia. Kruskal-Wallis test was used to compare the 3 groups in terms of Pain Score (NRS) at each of the time points. Chi-square test was used to compare quantitative data. *P* < .05 was considered statistically significant.

## Results

A total of 100 patients were assessed for eligibility, out of which 96 met the inclusion criteria. Ninety patients were included in final data analysis (Figure 1). Ten patients were excluded due to various reasons like patient refusal, conversion to open cholecystectomy, peritoneal biliary spillage, and surgery duration extended beyond 1 hour. There were no significant differences between the groups with respect to age, weight, and gender (Table 1).

Patients in the placebo group S reported higher NRS scores than the other study groups in the immediate PO period up to 6 hours PO. After that (at 8- and 12-hour measurement) placebo group S had lower NRS score due to earlier administration of rescue opioid dose. Nebulization of ropivacaine with or without nalbuphine had no significant difference in NRS scores at all time intervals. There was no difference in NRS score at 24 hours PO in all 3 groups (*P*  = .060) (Table 2).

Time to the first rescue analgesic after the surgery was the least in placebo group S with no significant difference between the experimental groups R and RN (Table 3). When the groups were compared in terms of total rescue analgesia (opioid) requirement, it was found that there was a statistically significant difference between the groups (*P* < .001). In the post-hoc analysis performed to determine the origin of the difference, the need for rescue analgesics in the placebo group was higher than in the other 2 experimental groups (*P* < .001, *P* < .001), but there was no difference between the experimental groups (*P* > .5) (Table 3).

Incidence of PO shoulder pain was higher in the placebo group S (3 out of 30 patients) compared to the ropivacaine nebulization groups (no shoulder pain in groups R and RN). Maximum cases of PONV were observed in the nalbuphine group RN (3 out of 30 patients). Group R had 2 out of 30 and group S had 1 out 30 patients. None of the patients enrolled in the study developed pruritus, respiratory depression, subcutaneous emphysema, sedation, or signs and symptoms of LAST or other notable adverse effects post-operatively.

## Discussion

In the current study, patients in the placebo group reported higher NRS scores than other study groups in the immediate PO period. Addition of nalbuphine did not cause any statistically significant improvement in pains scores as compared to ropivacaine administered alone. In addition to the currently studied technique, there are various new regional anaesthesia techniques that have been shown to be effective in minimally invasive surgeries for pain relief. These include erector spinae plane block, serratus intercostal plane block, oblique subcostal TAP, and quadratus lumborum block.^12-15^

Local anaesthetics, with or without opioids, are frequently being administered into the peritoneal cavity during minimally invasive procedures such as LC and gynecological laparoscopy by either instillation or nebulization.^6,7,16-18^ Nebulization offers a more homogenous spread of the drug intraperitoneally, as compared to instillation.^7^ Ultrasonic nebulizers which have been used in the current study, use high-frequency sound waves to generate aerosol particles of comparatively more uniform sizes of 1-5 microns compared to 4-10 microns from the conventional atomizers. The concerns with intraperitoneal nebulization are fogging and poor visibility in the cavity which may hamper the surgeon’s view limiting its use intraoperatively.^6,11^ Thus, in the current study it was used only at the end of the procedure. There is also a concern of increased systemic absorption of the drug through peritoneal surface. However, studies have shown the serum drug levels of local anaesthetics like ropivacaine to be well below toxic levels.^19^

In a double-blind, randomized, placebo-controlled trial by Ingelmo et al^11^ studying efficacy of intraperitoneal nebulization of ropivacaine (3 mL, 1%) for pain control after LC, it was concluded that ropivacaine nebulization before or after surgery reduced PO pain and referred shoulder pain, along with reduced morphine requirement and earlier mobility among patients who receive intraperitoneal ropivacaine nebulization. Similar findings were reported by Somaini et al^1^ using ropivacaine (3 mL, 1%). In the current study also, similar results were reported with ropivacaine ultrasonically nebulized at end of surgery (with or without nalbuphine) showing lower pain scores compared to placebo.

In the current study, no difference was found by addition of nalbuphine to ropivacaine. A study by Singh et al^20^ in LC patients with drug instillation in the peritoneal cavity showed decreased PO pain scores in the nalbuphine group. Also, a study by Bhatia et al^8^ showed better pain relief with fentanyl-ropivacaine intraperitoneal nebulization combination compared to nebulized ropivacaine alone. Recent studies on pre-emptive intravenous nalbuphine use in laparoscopic surgeries have also shown promise. It reduced the PO visceral pain and supplemental analgesic use^21^ and was also useful in ameliorating PO hyperalgesia induced by high-dose remifentanil use in LC.^22^

In 2015, Liu et al^2^ studied the combined usage of intraperitoneal and incisional ropivacaine for assessing pain severity after LC. Total of 160 patients were enrolled in this study. The main finding of this trial was that intraperitoneal and incisional ropivacaine (0.75%, 10 mL each) at the end of the LC significantly reduced the time of PACU (post-anaesthesia care unit) stay, PO dynamic pain, cumulative morphine requirements, and incidence of PONV. In the current study, lesser volume (8 mL) of 0.65% ropivacaine (52.5 mg) was used with similar efficacy, although no difference was observed in pain scores or total requirement of opioids by addition of nalbuphine to ropivacaine. Time to first rescue dose as well as total opioid requirement with ropivacaine use was significantly less compared to placebo and in keeping with the above study. Another dose finding and pharmacokinetic study concluded there was no enhancement in analgesic efficacy with higher doses (100, 150 mg) of nebulized ropivacaine compared to 50 mg during LC.^18^

In a study on PO analgesia after laparoscopic ovarian cyst resection comparing intraperitoneal nebulization and peritoneal instillation of ropivacaine, it was concluded that nebulization of ropivacaine (15 mL, 1%) prevented the use of morphine in a significant number of patients and reduced PO pain during the first few hours after surgery.^18,23^ In the current study also, lower pain scores were observed in the ropivacaine groups compared to placebo (immediate to 4 hours PO). From 8 to 12 hours PO, as rescue analgesia was provided in placebo groups earlier, the difference in pain scores between the 3 groups diminished. In the current study, total opioid consumption (fentanyl) was significantly higher in the placebo group compared to the ropivacaine and combined nalbuphine group similar to what was observed in the above-mentioned study.

In terms of side effects in the current study, no major adverse event was noted in any of the groups. Incidence of PONV was similar in all groups, in contrast to the study by Liu et al^2^ where ropivacaine had a lower incidence of PONV. This could be possibly due to the fact that in the current study, the sample was not adequate for the objective of detecting significant differences in the occurrence of PONV and cannot be extrapolated to the general population. No other opioid side effect such as respiratory depression was observed in any of the groups.

The limitation of current study: results cannot be extrapolated to a significant proportion of such cases such as long-duration surgeries, peritoneal spillage, and so on (which were excluded from the current study). Patient’s serum concentrations of study drugs could not be measured due to practical difficulties and/or non-feasibility. Further studies may be required to analyze safety concerns. Precautions taken in our study are keeping the doses of study drugs within permissible safety limits.^24^

To conclude, ropivacaine in a dose of 52.5 mg (0.65% in current study), nebulization is more effective than placebo for PO pain relief after LC. Addition of nalbuphine to ropivacaine nebulization does not significantly improve pain relief (NRS scores) after LC. Intraperitoneal ropivacaine nebulization has no significant adverse effect as compared to placebo. There was no adverse effect on the addition of nalbuphine to ropivacaine for intraperitoneal nebulization as compared to ropivacaine alone.

## Figures and Tables

**Table 1.** Demographic Data of the 3 Study Groups

**Parameters**	Group S (n = 30)	Group R (n = 30)	Group RN (n = 30)	*P*
**Age (Years)**	40.37 ± 14.44	43.60 ± 9.69	41.30 ± 11.66	.568^*^
**Gender** **Male** **Female**	7 (23.3%) 23 (76.7%)	7 (23.3%) 23 (76.7%)	7 (23.3%) 23 (76.7%)	1.000**#**
**Weight (kg)**	59.93 ± 9.62	58.20 ± 10.12	57.07 ± 10.08	0.462**$**

*One-way ANOVA; #Chi-squared test; $Kruskal–Wallis test; *P* < .05 is significant.

Group S (placebo), R (ropivacaine only), RN (ropivacaine and nalbuphine).

**Table 2.** Comparison of Post-operative NRS Pain Score in the Three Study Groups

**NRS score median (IQR)**	**Group S**	**Group R**	**Group RN**	*P* *			
**S, R, RN**	**S, R**	**S, RN**	**R, RN**
Immediate PO	2 (1-2)	1 (0-1)	1 (0-1)	.003	.009	.009	1.000
1 hour PO	3 (2-3)	2 (1-2)	2 (1-2)	<.001	<.001	<.001	1.000
2 hours PO	4 (3-4)	2 (2-3)	2.5 (2-3)	<.001	.001	.002	.998
4 hours PO	3 (3-4)	3 (3-3)	3 (3-3)	.238	-	-	-
8 hours PO	3 (2-3)	3 (3-4)	3 (3-4)	.002	.005	.006	1.000
12 hours PO	2 (2-2)	3 (2-3)	2.5 (2-3)	.002	.007	.006	1.000
24 hours PO	1 (1-1)	1 (0-1)	1 (0-1)	.060	-	-	-

PO, post-operative; IQR, inter-quartile range; NRS, numerical rating scale.

*Kruskal–Wallis test with post hoc analysis *P* < .05 is significant.

Group S (placebo), R (ropivacaine only), RN (ropivacaine and nalbuphine).

**Table 3.** Comparison of Analgesic Requirement Profile in the Three Study Groups

**Parameters**	**Group S**	**Group R**	**Group RN**	*P* *			
**S, R, RN**	**S, R**	**S, RN**	**R, RN**
**Time of first opioid requirement (hours)**	2.80 ± 2.07	6.73 ± 3.50	6.87 ± 3.63	<.001	<.001	<.001	.999
**Total opioid requirement (μg kg-1) in first 24 hours**	2.50 ± 0.82	1.30 ± 0.53	1.23 ± 0.50	<.001	<.001	<.001	.969

*Kruskal-Wallis test with post hoc test; *P* < .05 is significant, Group R (placebo), R (ropivacaine only), RN (eopivacaine and nalbuphine).
